# Inconsistency in trauma reporting: role of PTSD, depression and psychological distress in a longitudinal study among healthcare workers

**DOI:** 10.1192/bjo.2026.10983

**Published:** 2026-03-10

**Authors:** Mariam El-Jamal, Josleen Al Barathie, Elie Karam

**Affiliations:** Institute for Development, Advocacy, and Applied Care (IDRAAC), Beirut, Lebanon; Department of Psychiatry and Clinical Psychology, Faculty of Medicine, Saint George University of Beirut, Lebanon; Department of Psychiatry and Clinical Psychology, Saint George Hospital University Medical Center, Beirut, Lebanon

**Keywords:** Inconsistent trauma reporting, PTSD, depression, memory exaggeration, memory alteration

## Abstract

**Background:**

Accurate trauma recollections are essential in legal and research contexts; however, studies frequently reveal significant inconsistencies in trauma reporting over time.

**Aims:**

To investigate the trauma-reporting patterns among healthcare workers (HCWs) following their exposure to the Beirut port blast.

**Method:**

This longitudinal study examined trauma memory alteration among 296 HCWs at 6 months (wave 3) and 2–2.5 years (wave 4) post-blast. Participants reported trauma exposure prior to the event, and probable post-traumatic stress disorder (PTSD) secondary to the Beirut port blast. Depression and psychological distress were analysed as potential predictors of memory alteration using multinomial models.

**Results:**

The majority of participants (72.4%) exhibited inconsistent trauma reporting, with 36.43% exaggerating and 35.71% diminishing their trauma accounts over time. Developing probable depression and screening positive for PTSD at wave 4 were predictors of memory exaggeration (respectively odds ratio 5.71, 95% CI: 1.19–27.32; odds ratio 8.04, 95% CI: 0.98–65.73), while remitted psychological distress was protective (odds ratio 0.08, 95% CI: 0.01–0.99). No significant predictors were found for memory diminishment.

**Conclusions:**

A substantial portion of HCWs exposed to the Beirut port blast demonstrated inconsistent trauma reporting, with mental health conditions such as depression and PTSD influencing memory exaggeration. These findings underscore the importance of considering memory reliability in trauma research, particularly in populations with mental health disorders and exposed to major disasters.

Researchers have long tried to unpack and dispel myths surrounding the function of memories in the human brain and what affects their alteration and distortion.^1,2^ A common product of memory distortions in legal contexts is eyewitness misidentification which unfortunately occurs in high rates and leads to false accusations and conviction of innocent individuals.^3–5^ Memory alterations not only invade legal contexts, but they also influence trauma-related research.

## Rates of inconsistent trauma reporting

Many studies have shown that individuals often provide inconsistent reports of their traumas when asked about them at different points in time. Hepp et al, for example, found that nearly 63% of the individuals they interviewed in 1993 inconsistently reported their traumas in their second interview in 1999, 6 years after the initial encounter.^6^ Similarly, Coleman et al found that of the 7466 individuals they interviewed in 1994/1995 and again in 2006/2007, 40% inconsistently reported their traumas within a 12-year interval.^7^ Moreover, in their systematic review and meta-analyses, Baldwin et al compared retrospective and prospective recollections of childhood maltreatment in seven studies.^8^ They found that the agreement between the two accounts was low, with a significant portion of individuals (52%) failing to retrospectively report maltreatment despite reporting it prospectively.^8^ Likewise, Francis et al also showed low agreement between self-reports of childhood adversities and objective reports (taken from official records or other official unbiased sources).^9^ As previously pointed out, the literature is rich with evidence regarding inconsistent trauma reporting among individuals over time.^10–13^ It is worth noting that methodological differences across studies, including variations in follow-up intervals, trauma definitions, measurement tools used (e.g. checklists) and the context in which trauma is assessed, can partially explain these discrepancies. Some studies may also differ in their use of prospective versus retrospective approaches, which can have a further impact on reporting consistency.

## Predictors of inconsistent trauma reporting

There are many factors that influence this inconsistency in trauma reporting. Some of the factors that predict under-reporting of certain adversities include social desirability bias, self-protective mechanisms and personality traits such as high agreeableness.^14,15^ Another important factor that has an impact on inconsistency in trauma reporting is the presence of psychopathologies and certain mental health disorders. The literature is replete with evidence showing that individuals with depression have a higher tendency to report and recall sad memories. For instance, in Fogarty’s 2-year longitudinal study, depressed individuals were significantly associated with recalling negative memories, and as their depression improved, they tended to remember fewer sad memories.^16^ Moreover, a more recent longitudinal study by Goltermann showed that states of depression can result in a slight increase in accounts of childhood adversities.^17^ Consistent with these findings, Coleman et al also found that developing depression was associated with an increased risk of reporting adverse childhood experiences that had not been previously reported.^7^ Besides depression, evidence shows that post-traumatic stress disorder (PTSD) also influences the recollection of trauma-related memories. Intrusive memories, which are key features in the diagnosis of PTSD, are disrupted, unorganised and sometimes involuntary recollections of traumatic events.^18,19^ This process can contribute to inconsistent reporting of certain traumas. For example, Roemer et al showed that veteran survivors tend to inflate their trauma accounts across time, and their intrusive PTSD symptoms were directly and positively associated with their memory alteration.^20^ Similarly, Southwick et al showed that nearly 88% of the 59 veterans interviewed inconsistently reported on their traumas in a 2-year period.^21^ The study showed that as the symptoms of PTSD increased, a significant increase in the exaggeration of traumatic events with time was observed.^21^ Other mental health conditions that are associated with inconsistency of trauma reporting include psychological distress, psychotic experiences and emotional and behavioural problems.^7,10^

## Current study’s scope and relevance

After the Beirut port blast on 4 August 2020, we followed-up healthcare workers (HCWs) who witnessed the explosion in Saint George Hospital University Medical Center (SGHUMC) owing to its very close proximity to the Beirut port. Our current study aims to explore the trauma-reporting patterns of these HCWs over time and identify the relevant associated variables. There are three main aspects of relevance of our study. First, unlike much of the existing literature about the inconsistency of trauma reporting, we do not focus on childhood adversities alone, rather we also examine other types of traumas – including traumas such as the death of a loved one, a major accident or personal physical illness. Second, our study investigates several psychopathologies and mental health issues as associated factors, including PTSD secondary to the Beirut port blast, depression and psychological distress. Most of the existing research examines these mental health conditions separately, with limited exploration of PTSD. Our inclusion of PTSD in the analyses represents a significant strength in our study. Finally, we could not find any research exploring trauma-reporting patterns and their associated factors among HCWs. Most of the literature focuses either on survivors of childhood abuse, combat veterans or the general community. Our study fills this gap by specifically focusing on HCWs, particularly during a time when research on their mental health is gaining more attention in the aftermath of the 2020 pandemic. The inclusion of HCWs is important for several reasons. First, this group remains largely overlooked in research – to our understanding, there has been no previous investigation into the patterns of memory alteration within this population. Second, in the global context of the pandemic, HCWs experience increased psychological pressure, which calls for more research. Finally, SGHUMC, the site of our study, experienced significant effects from the Beirut port blast, with many HCWs directly exposed and injured. This situation creates a particularly relevant group for examining trauma-recollection patterns, as they are both victims and frontliners.

## Method

### Study design, setting and participants

On 4 August 2020, Lebanon witnessed one of the largest non-nuclear blasts in modern history.^22^ The explosion resulted in more than 200 fatalities, 6000 injuries and 300 000 displacements.^23^ Owing to its close proximity to the port, SGHUMC was severely hit by the blast, and its employees were substantially exposed to the explosion. Given this context, it is important to mention that most HCWs in our sample lived in close proximity to the hospital or resided in on-site dormitories, as commuting from distant regions is relatively uncommon in Lebanon. Consequently, the majority of participants experienced direct exposure to the Beirut port blast. The current longitudinal study followed HCWs at SGHUMC, aged 18 years and above for 2–2.5 years at 4 different intervals. The first two waves were part of a national initiative following the Beirut port blast while the third and fourth waves were part of an ongoing multinational study focused on HCWs and COVID-19.^24^

The study commenced 9–15 days following the Beirut port blast in 2020 – between 13 August and 19 August 2020 – when the hospital administration urged all its workers to undergo polymerase chain reaction (PCR) testing due to the accumulating rates of COVID-19 cases following the chaotic situation of the blast. This first wave of data collection happened in person using self-filled questionnaires in the PCR screening area, and 570 participants managed to complete the survey after consenting orally, which was witnessed and formally recorded.

Later waves of data collection were conducted 21–27 days, 6–7 months and 2–2.5 years after the blast. The data were collected online by disseminating survey links through mass emails to the hospital’s employees including clinical, administrative and supportive staff. Although the main focus of the study was to follow up on participants across the four waves, we also recruited new participants in each interval to maximise our sample size. Therefore, the final samples of waves 2, 3 and 4 were 733, 808 and 524 respectively. More information about time and mode of assessment, total number of participants and the response rate of each wave can be found in the Tables S1 and S2 in the supplementary material available at https://doi.org/10.1192/bjo.2026.10983.

In this study, questions pertaining to prior traumas were asked twice in waves 3 and 4 where 808 and 524 individuals were recruited respectively. Among those, 296 participants were followed up on in both waves, and they will be the focus of our study.

The authors assert that all procedures contributing to this work comply with the ethical standards of the relevant national and institutional committees on human experimentation and with the Helsinki Declaration of 1975, as revised in 2013. All procedures involving human subjects were approved by the Institutional Review Board (IRB) committee of the SGHUMC Faulty of Medicine, University of Balamand, Lebanon, which is registered with the US Office of Human Research Protections (OHRP) in the Department of Health and Human Services. The approval number of the study is IRB-REC/O/ 019-20/1120. Informed written consent was taken from participants before they started filling in the surveys. These consents were later emailed to the respective participants for full transparency.

### Instruments and measures

In addition to sociodemographic information (age, gender and profession), the survey included a PCL-5 proxy DSM-5 diagnosis of PTSD, a General Health Questionnaire (GHQ-12) and a Patient Health Questionnaire (PHQ-9). Additionally, we asked participants questions about their trauma exposure prior to the Beirut blast.

#### PCL-5

In both waves 3 and 4, we used the PTSD checklist for DSM-5 (PCL-5), a 20-item self-report questionnaire, to determine rates of probable PTSD secondary to the Beirut port blast. Following the PCL-5 instructions, the items were to be answered in reference to the Beirut port blast as the index trauma to ensure that participants rated their symptoms specifically in relation to this event. Participants rated symptoms on a scale from 0 (not at all) to 4 (extremely), aligning with DSM-5 criteria.^25^ Symptoms rated at a moderate level (2) or higher were considered endorsed. A DSM-5 proxy diagnosis necessitates meeting specific criteria: at least one item from Intrusion (Criterion B), one from Persistent Avoidance (Criterion C), two from Negative Alterations in Cognitions and Mood (Criterion D) and two from Arousal and Reactivity (Criterion E).

#### GHQ-12

In both waves 3 and 4, we used the General Health Questionnaire-12 (GHQ-12), a widely utilised 12-item self-report measure, to determine the rates of psychological distress in our sample.^26^ The GHQ-12 items are designed to detect non-psychotic psychiatric disorders in community settings. Each item is scored on a 4-point Likert scale, typically ranging from 0 to 3, with higher scores indicating greater psychological distress. The total score can range from 0 to 36, with a cut-off of 12 used to distinguish between ‘cases’ and ‘non-cases’ of psychological distress. The psychometric properties of the GHQ-12 have been well-documented, demonstrating good reliability and validity across diverse populations.^26,27^

#### PHQ-9

In both waves 3 and 4, we used PHQ-9, a 9-item self-report tool to determine rates of probable depression among participants in the past 2 weeks. Each question is assigned a score from 0 to 3, where 0 signifies ‘not at all’ and 3 denotes ‘nearly every day.’ After summing the 9 items of PHQ-9, a total score ranging from 0 to 27 is obtained for each participant. A cut-off score of 10 was used for PHQ-9, which yields a specificity of 0.89 and a sensitivity of 0.85.^28^

#### Prior traumas

In waves 3 and 4, 6–7 months and 2–2.5 years following the Beirut port blast, respectively, we asked participants specific binary questions about specific traumas they had encountered before the 2020 Beirut port blast. These questions ranged from childhood adversities like neglect, parental physical abuse to experiences of sexual abuse. Additionally, participants were asked whether they had witnessed any of the following traumas prior to the 2020 blast: the death of a loved one, personal physical injuries, involvement in major accidents, serious physical illness of a loved one or exposure to war events.

#### Memory alteration variables

To assess how the trauma-related memory of each participant was altered from wave 3 to wave 4, we first summed the binary trauma-related questions of each wave. These included questions about childhood adversities (namely neglect, whether or not they were hit by their parents and exposure to sexual abuse), death of a loved one, major accident, personal physical illness, major physical illness of a loved one and exposure to war before the Beirut port blast of 2020. The difference between these variables was generated (sum of trauma-related questions of wave 4 – sum of trauma-related questions of wave 3). Thus, we obtained the following:

- (a) When the individual failed to report the trauma in wave 4 but had reported it in the previous wave, this individual yielded a negative difference, and we considered his/ her memory of the trauma to have diminished with time.
- (b) When an individual failed to report the trauma in wave 3 and reported it when he/she was asked again in wave 4, the participant had a positive difference across the waves, and we considered his/her memory to have been exaggerated with time.
- (c) When the difference in reporting was zero across waves, we considered that there was no memory alteration observed in that individual.

### Statistical analyses

Descriptive statistics of the sample were summarised using frequencies and percentages for categorical variables and means and standard deviations for continuous variables. Differences across categorical and continuous variables were evaluated using chi-squared tests and analysis of variance, respectively.

Simple and multiple multinomial analyses were conducted for a memory alteration variable (which includes diminishment and exaggeration with no change as reference category). Variables that achieved statistical significance at the level of the simple multinomial analyses were included in our final model, along with confounders such as age, gender and profession. We also included profession to distinguish between the type of HCWs. Odds ratios, 95% CI and *p*-values were reported. All analyses were performed using Stata 17 for Windows (StataCorp LLC, College Station, Texas, USA; https://www.stata.com).

## Results

### Descriptive statistics

In Table 1, we present the distribution of memory alteration patterns along with the associated characteristics of the study participants. Questions about the death of a loved one (44.36%) and exposure to war (66.36%) exhibited the highest rates of memory alteration from waves 3 to 4. For a detailed breakdown of memory alteration status for each trauma question, including the proportion of individuals who experienced diminishment, exaggeration or no change in memory from wave 3 to 4 please refer to Table S3 in the supplementary material.

**Table 1:** Descriptive statistics of memory alteration patterns

Category	No change, *n* (%)	Diminishment, *n* (%)	Exaggeration, *n* (%)	*p*-value^a^
Total	78 (27.86)	100 (35.71)	102 (36.43)	
Age, mean ± s.d.	40.17 ± 12.15	42.85 ± 12.02	39.25 ± 12.22	0.623^b^
Gender				0.723
Female	58 (26.61)	81 (37.16)	79 (36.24)	
Male	18 (30)	19 (31.67)	23 (38.33)	
Profession				0.654
Non-clinical	26 (29.55)	28 (31.82)	34 (38.64)	
Clinical	52 (27.08)	72 (37.5)	68 (35.42)	
PTSD (W3)				0.533
No	57 (28.64)	67 (33.67)	75 (37.69)	
Yes	21 (25.93)	33 (40.74)	27 (33.33)	
PTSD (W4)				<0.001
No	77 (30.56)	93 (36.9)	82 (32.54)	
Yes	1 (3.57)	7 (25)	20 (71.43)	
PTSD trend				0.002
Never PTSD	57 (31.15)	64 (34.97)	62 (33.88)	
Consistently PTSD	1 (8.33)	4 (33.33)	7 (58.33)	
Remitted PTSD	20 (28.99)	29 (42.03)	20 (28.99)	
Developed PTSD	0 (0)	3 (18.75)	13 (81.25)	
Depression trend				0.046
Never depressed	56 (30.6)	67 (36.6 1)	60 (32.79)	
Consistently depressed	1 (25)	1 (25)	2 (50)	
Remitted depression	6 (25)	9 (37.5)	9 (37.5)	
Developed depression	2 (8)	6 (24)	17 (68)	
Psychological distress trend				0.014
Never distressed	3 (23.08)	1 (7.69)	9 (69.23)	
Consistently distressed	29 (25.44)	42 (36.84)	43 (37.72)	
Remitted distress	5 (71.43)	1 (14.29)	1 (14.29)	
Developed distress	26 (25.49)	44 (43.14)	32 (31.37)	

PTSD, post-traumatic stress disorder; W3, Wave 3; W4, Wave 4.

a. *p*-value corresponding to chi-squared performed between variable containing exaggeration, diminishment and no change with each corresponding variable.

b. Analysis of variance.

Among the participants, 78 (27.86%) individuals experienced no change in memory, while 100 (35.71%) experienced memory diminishment and 102 (36.43%) experienced memory exaggeration. The mean age varied across groups, with those experiencing diminishment being slightly older (42.85 ± 12.02 years) compared with those with no change (40.17 ± 12.15 years) or exaggeration (39.25 ± 12.22 years). Gender distribution showed minor variations, with females comprising slightly higher proportions in the diminishment (*n* = 81, 37.16%) and exaggeration (*n* = 79, 36.24%) groups compared with the no change group (*n* = 58, 26.61%). Similarly, clinical professionals were slightly over-represented among individuals with memory diminishment (*n* = 72, 37.5%) and exaggeration (*n* = 68, 35.42%) compared with those with no change in their memory (*n* = 52, 27.08%). None of the previous variables achieved statistically significant differences.

As for the variables that were statistically associated with the outcome, the trend of probable PTSD secondary to the Beirut port blast showed that all participants who developed PTSD at wave 4 either had memory diminishment (*n* = 3, 18.75%) or memory exaggeration (*n* = 13, 81.25%). Probable PTSD at wave 4 was higher among individuals experiencing memory exaggeration (*n* = 20, 71.43%) compared with those with no change (*n* = 1, 3.57%) or diminishment (*n* = 7, 25%). Similarly, most participants who developed probable depression from wave 3 to wave 4 experienced memory exaggeration (*n* = 17, 68%) compared with the other groups. Finally, the majority of the study participants with remitted distress experienced no change in the recollections of their traumas (*n* = 5, 71.43%). For a detailed overview of the descriptive statistics, kindly refer to Table 1.

### Simple and multiple multinomial analyses

In Table 2, we present both the simple and multiple multinomial analyses examining each memory diminishment and exaggeration in comparison with the absence of memory alteration. At the simple multinomial level, none of the studied characteristics demonstrated a significant association with diminishment. However, for memory exaggeration, developing probable depression (odds ratio 7.93, 95% CI: 1.75–35.9, *p*-value: 0.007) emerged as a significant risk factor, similarly remitted distress (odds ratio 0.07, 95% CI: 0.01–0.82, *p*-value: 0.035) appeared as a significant protective factor.

**Table 2:** Bivariate and multivariate analyses for each outcome diminishment and exaggeration

Category	Diminishment versus no change in memory						Exaggeration versus no change in memory					
	OR	95% CI	*p*-value	aOR	95% CI	*p*-value	OR	95% CI	*p*-value	aOR	95% CI	*p*-value
Age	1.02	(0.99–1.04)	0.157	1.02	(0.99–1.05)	0.268	0.99	(0.97–1.02)	0.614	0.99	(0.96, 1.02)	0.537
Gender												
Female	1.00			1.00			1.00			1.00		
Male	0.76	(0.37–1.56)	0.451	0.60	(0.24–1.52)	0.278	0.94	(0.46–1.9)	0.859	0.83	(0.34, 2.05)	0.693
Profession												
Non-clinical	1.00						1.00					
Clinical	1.29	(0.37–1.56)	0.443				1.00	(0.37–1.56)	1			
Depression variation												
Never depressed	1.00			1.00			1.00			1.00		
Consistently depressed	0.84	(0.05–13.67)	0.9	0.83	(0.05–14.25)	0.895	1.87	(0.16–21.16)	0.614	1.30	(0.1, 16.85)	0.843
Remitted depression	1.25	(0.42–3.74)	0.685	0.98	(0.27–3.61)	0.98	1.40	(0.47–4.19)	0.574	0.88	(0.23, 3.35)	0.852
Developed depression	2.51	(0.49–12.92)	0.272	2.12	(0.39–11.46)	0.383	7.93	(1.75–35.9)	0.007^*^	5.65	(1.17, 27.17)	0.031^*^
PTSD variation												
Never PTSD	1.00			1.00						1.00		
Consistently PTSD	3.56	(0.39–32.81)	0.262	1.18	(0.1–14.42)	0.898	6.44	(0.77–53.93)	0.086	3.98	(0.42, 37.63)	0.228
Remitted PTSD	1.29	(0.66–2.53)	0.456	1.27	(0.56–2.88)	0.571	0.92	(0.45–1.88)	0.818	1.02	(0.42, 2.43)	0.973
Developed PTSD	Omitted			Omitted			Omitted			Omitted		
Psychological distress variation												
Never distressed	1.00			1.00			1.00			1.00		
Consistently distressed	4.34	(0.43–43.86)	0.213	3.79	(0.35–41.34)	0.275	0.49	(0.12–1.98)	0.32	0.43	(0.09, 1.98)	0.275
Remitted distress	0.60	(0.03–13.58)	0.748	0.52	(0.02–12.36)	0.688	0.07	(0.01–0.82)	0.035^*^	0.08	(0.01, 1.07)	0.056
Developed distress	5.08	(0.5–51.38)	0.169	4.14	(0.39–43.86)	0.239	0.41	(0.1–1.67)	0.214	0.32	(0.07, 1.46)	0.142

OR, crude odds ratio of simple multinomial analyses; aOR, adjusted odds ratio of multiple multinomial analyses; PTSD, post-traumatic stress disorder.

* Indicates variables that were significant (*p*-value <0.05).

Consequently, depression trend, and psychological distress were all incorporated into the multiple multinomial analyses, all while controlling for gender and age. In the case of diminishment, none of the variables reached significance in the multiple multinomial analyses. In the case of memory exaggeration, however, probable depression remained a significant risk factor in the final model. Specifically, individuals who developed probable depression were at a higher risk of exaggerating their memories compared with those who were never depressed in either wave 3 or wave 4 (odds ratio 5.65, 95% CI: 1.17–27.17, *p*-value: 0.031). Moreover, remitted psychological distress remained a protective factor (odds ratio 0.08, 95% CI: 0.01–1.07, *p*-value: 0.056), albeit with borderline significance.

### Supplementary analyses

In our main analyses, the variable PTSD trend was significantly associated with the outcome in the multinomial analyses, although none of the specific categories of the variables were significant. To further explore this, we conducted additional analyses with probable PTSD secondary to the Beirut port blast assessed cross-sectionally at wave 3 and wave 4. The analyses can be found in Table S4 in the supplementary material. We found that both trends in probable depression and psychological distress maintained their association with memory exaggeration compared with no change in memory (developed probable depression: odds ratio 5.71, 95% CI: 1.19–27.32, *p* = 0.029; remitted distress: odds ratio 0.08, 95% CI: 0.01–0.99, *p* = 0.049). Interestingly, PTSD at wave 4 emerged as a significant predictor in the simple multinomial regression (odds ratio 18.78, 95% CI: 2.46–143.33, *p* = 0.005) and as a borderline significant predictor in the full model (odds ratio 8.04, 95% CI: 0.98–65.73, *p* = 0.052). Similar to the initial analyses, none of the explored variables explained memory diminishment.

## Discussion

### Prevalence of inconsistency in trauma reporting

In this study, we explored the prevalence and predictors of the inconsistency of reporting trauma-related questions among HCWs who were exposed to the Beirut blast. The majority of our sample (72.4%) inconsistently answered the trauma-related questions. Most of them either exaggerated (36.43%) their trauma reports or diminished (35.71%) their accounts.

The rate of inconsistency in our sample aligns with the patterns found in the literature. Studies following veterans and trauma survivors have reported inconsistency rates in trauma reporting ranging from 50 to 89%.^12,21,29^ Moreover, in community studies such as Hepp et al’s, a 63.9% inconsistency rate was revealed when participants were asked the same trauma-related questions after 6 years.^6^

Age and the focus on childhood adversities also play a significant role in these findings. Previous studies have shown that older age is a significant risk factor for memory distortion, which contributes to higher inconsistency rates. For example, Burns et al found a lower rate of 16.3% inconsistency in a cohort of children and adolescents, where participants inconsistently reported their trauma after initially endorsing it in their first interview.^10^ This lower rate can be attributed to the fact that the study focused exclusively on childhood maltreatment and involved a younger sample. Similarly, Coleman et al observed an inconsistency rate of 40% after a 12-year follow-up in a community sample.^7^ The lower rates compared with our study are likely because their questions were limited to childhood maltreatment alone. The narrower focus and younger age range in these studies help explain the disparity in inconsistency rates compared with our findings.

### Predictors of trauma exaggeration

Our findings show that probable PTSD secondary to the Beirut port blast, probable depression and psychological distress are all significantly associated with exaggeration or inflation of trauma endorsement. This is in line with the literature, which states that psychopathologies and mental health outcomes predict memory distortion. There are many explanations for these findings.

### PTSD and memory inflation

We know from the literature that PTSD is commonly associated with memory inflation of traumatic events,^20,21,30,31^ and this can be explained in two ways.

First, a key feature of PTSD diagnosis is intrusive memories.^18,19^ These memories can lead to disrupted and involuntary recollection of traumatic experiences, fostering the inflation of traumatic memories.^20^

Second, among the 13 individuals who developed PTSD at wave 4 and showed memory exaggeration, the diagnosis can be classified as delayed PTSD, occurring two years after the blast. Delayed PTSD has been shown to predict memory inflation,^32^ offering an additional explanation for our findings alongside intrusive symptoms.

In our sample, the variable corresponding to the trend of probable PTSD diagnosis could not fully account for the inflation of traumatic memory endorsement. This limitation stems from the small sample size and the uniform experience among participants who developed PTSD by wave 4. Specifically, all individuals in this subgroup exhibited either memory exaggeration or distortion; none reported no change in their trauma reporting. Consequently, the lack of variability in our outcome variable prevented us from drawing definitive conclusions regarding the influence of PTSD development. However, supplementary analysis revealed that only PTSD at wave 4 was associated with the inflation of traumatic memory reporting. Therefore, it is possible that the observed association is primarily driven by the onset of PTSD at wave 4.

Hence, despite being obscured in the main analyses due to limited sample size and the lack of variability in the variable under study, our results show that developing PTSD by wave 4 predicts memory exaggeration. This can partially be explained by Ehlers et al’s cognitive model of PTSD which states that the persistence of the disorder is often associated with how serious and current the threat of the trauma is processed.^33^ This in part is explained by the disturbance of autobiographical memory, including selective attention to threat and memory amplification which is evident in our sample.

A further mechanism worth considering is priming, which the literature describes as the activation of trauma-related cognitive networks in response to reminders of the event.^33,34^ Empirical studies show that individuals with PTSD exhibit heightened sensitivity to trauma cues, and such cues can trigger symptom reactivation and influence memory reconstruction. Because our study assessed trauma repeatedly across waves, within a context where the Beirut blast remained highly salient, participants may have been repeatedly primed through the survey itself. This repeated activation could bias the consolidation and retrieval of trauma memories towards inflation. Moreover, the directionality between memory and PTSD is unlikely to be linear; PTSD may increase the likelihood of exaggerated recall, while exaggerated recall may reinforce trauma-related threat appraisals, thereby maintaining or intensifying PTSD symptoms. Integrating a priming perspective therefore provides a more comprehensive understanding of the cognitive processes that may underlie, partially, the inconsistency and inflation observed in our sample.

### Depression, psychological distress and trauma memory inflation

We have shown before in a nationally representative sample of Lebanese participants that the multiple stressors that the country has witnessed (including the financial meltdown and the global pandemic) were the main drivers of the observed high rates of mental health disorders in the sample.^35^ This is relevant to our findings because the majority of our sample had psychological stress in either wave 3 or 4. We believe that this increased rate of distress alongside the observed rates of depression also contributed to the exaggerated trauma responses in our sample.

Research shows that both negative mood and clinical depression bias the effect of memory recollection – this is known as the mood congruency theory.^11^ Particularly, psychological distress and depression induce the recollection of sad memories. Depressed individuals are more likely to experience recall bias, particularly for negative information, which influences the increased reporting of trauma-related memories.^36^

Our findings add to the literature on trauma-related cognitive processes by suggesting that trauma memory may be influenced by the presence and severity of PTSD symptoms. These processes are not fully captured by general mood-congruent memory effects, but are instead grounded in trauma-specific schemas that may bias the encoding and retrieval of prior adverse experiences, particularly when contextual cues resemble aspects of the index trauma.^37^

Also, the directionality of the observed associations warrants careful consideration. While our cross-sectional findings suggest that PTSD symptoms may have an impact on the recall, it is also plausible that memory inflation or reinterpretation over time contributes to the persistence or intensification of PTSD symptoms. The relationship is likely bidirectional, and future longitudinal or experimental studies would be required to disentangle these pathways.^38^

This aligns with our findings. Our sample demonstrated that individuals who developed depression by wave 4 were at a higher risk of inflating their trauma-related memories. This finding is similar to those of Coleman et al,^7^ and others who have shown that the development or worsening of depressive symptoms over time predicts the exaggeration of traumatic memories.^16^

### Generalisation and psychopathology

Another explanation for the observed inflation of traumatic events in our sample is the overgeneralisability associated with psychopathologies.^36^ According to the literature, individuals with psychopathologies are more likely to generalise their reports of traumatic events. This tendency to overgeneralise could also be contributing to the inflated reports observed in our study.

### Lack of predictors for memory diminishment

We should point out that in our study, none of the predictors explained memory diminishment. This is not an unusual finding since many have also found that after being exposed to a major stressful event – which in our case is the Beirut port blast – individuals tend to exaggerate their stressful events as time passes.^20,21,30,31^ This can explain why none of our variables predicted memory diminishment.

### Study limitations

Our study has several limitations that should be acknowledged. First, we did not collect data on the age at which participants experienced their trauma. Consequently, we could not assess how the timing of the traumatic event influenced the consistency of recall. Second, the trauma-related memories reported by HCWs in our study were self-reported, and we did not have an assessment to verify these reports. This is a significant limitation because research indicates that discrepancies between objective and subjective reports of maltreatment and trauma are common.^8,9,12^ Although classifying a memory as definitively ‘true’ is not always accessible, we encourage objective verification of reported incidents to reduce inconsistency in trauma reporting. This is especially true for self-reported measures that can suffer due to the participant’s potential inattention during the administration of the survey. Hence, without objective verification, we cannot determine the accuracy of the reported traumas, which has an impact on the validity of our findings regarding memory diminishment and exaggeration. Third, while we included a question assessing whether participants were physically injured in the explosion (as an objective indicator of threat), we did not assess their subjective perception of life threat at the time of the event. This may influence the encoding and recall of traumatic memories. Lastly, we did not control for several behavioural factors that can influence the consistency of trauma reporting. These include self-esteem, chronic stress, mastery, psychotic episodes in addition to treatment-seeking options.^7,10^ The lack of control for these variables may have affected the results, as these factors can also contribute to variations in trauma recall.

### Strengths and recommendations

One of the strengths of our study is that we controlled for age. This is particularly important as older age is associated with memory diminishment. By accounting for age, we minimised its potential confounding effect on the consistency of trauma recall. Moreover, we also controlled for social desirability bias by ensuring that the responses were anonymous. Participants were assured that their identities would not be disclosed since the surveys were sent via email. This approach likely encouraged more honest and accurate reporting of trauma-related memories.

For future studies, it is recommended to ask about the severity and onset of the trauma. Collecting this data would allow researchers to objectively trace the trauma over time and better understand its impact on memory consistency. Moreover, to address the limitation of relying on self-reported trauma, future research should incorporate objective assessments to verify the reported traumas. This could involve cross-referencing self-reports with medical records or using validated trauma assessment tools.

### Research implications

Our study has important implications for trauma research among disaster survivors such as military personnel, war refugees or emergency responders: inconsistencies in self-reported trauma may reflect dynamic psychological processes rather than measurement error alone. Researchers should therefore interpret changes in trauma narratives with caution, consider repeated assessments over time and incorporate mental health indicators when analysing self-reported trauma data. This approach can improve the validity of trauma research findings and ensure more accurate relationships between trauma exposure and mental health outcomes.

In conclusion, this study provides insight into the variability of trauma reporting among HCWs following the Beirut blast, where over 70% of participants exhibited inconsistency in their accounts. The strong correlation between trauma exaggeration and mental health conditions like PTSD and depression suggests that these psychological factors may distort trauma memories, leading to inflated reports over time. Our findings align with existing literature, reaffirming the need for clinicians and researchers to be aware of the potential biases in self-reported trauma, particularly in individuals with underlying psychopathologies.

## Supporting information

El-Jamal et al. supplementary material El-Jamal et al. supplementary material

## Data Availability

The data that support the findings of this study are available from the corresponding author, E.K., upon reasonable request.

## Supplementary material

The supplementary material is available online at https://doi.org/10.1192/bjo.2026.10983
