# Supplementary material for: Inconsistency in trauma reporting: role of PTSD, depression and psychological distress in a longitudinal study among healthcare workers
Source: BJPsych Open. 2026 Mar 10;12(2):e84. doi: 10.1192/bjo.2026.10983 (PMC13107334; doi:10.1192/bjo.2026.10983)
Supplement: El-Jamal et al. supplementary material [file S2056472426109831sup001.docx]

Table S1. Description of study participants at Baseline

|  | **Wave 1 (9-15 days)** | **Wave 2 (21-27 days)** |
| --- | --- | --- |
| **N (Response Rate)** | 570 (NA) | 730 (38%) |
| **Age (Mean± SD)** | 34.05 ± 11.14 | 34.66±11.31 |
| **Gender** |  |  |
| Males | 197 (34.62%) | 249 (34.06%) |
| Females | 372 (65.38%) | 482 (65.94%) |
| **Profession** |  |  |
| Non-Clinical | 139 (26.23%) | 232 (32.58%) |
| Clinical | 391 (73.77%) | 480 (67.42%) |

Table S2. Description of Study participants at Follow-ups 1 and 2

|  | **Wave 3 (6-7 months)** | **Wave 4 (2-2.5 years)** |
| --- | --- | --- |
| **N (Response Rate)** | 808 (41.8%) | 524 (40.5%) |
| **Age (Mean± SD)** | 36.90 ± 12.36 | 41.86 ± 12.87 |
| **Gender** |  |  |
| Males | 236 (29.28%) | 151 (28.98%) |
| Females | 570 (70.72%) | 370 (71.02%) |
| **Profession** |  |  |
| Non-Clinical | 200 (24.94%) | 139 (26.53%) |
| Clinical | 602 (75.06%) | 385 (73.47%) |
| **PTSD Diagnosis** |  |  |
| No PTSD | 587 (72.65%) | 466 (88.93%) |
| PTSD | 221 (27.35%) | 58 (11.07%) |
| **Depression** |  |  |
| No Depression | 540 (83.2%) | 401 (85.87%) |
| Depression | 109 (16.80%) | 66 (14.13%) |
| **Psychological Distress** |  |  |
| No Distress | 291 (43.96%) | 37 (7.99%) |
| Distress | 371 (56.04%) | 426 (92.01%) |

| Table S3. Individual Trauma-Related Question Differences Across Waves | | | | |  |  |  |  |
| --- | --- | --- | --- | --- | --- | --- | --- | --- |
|  | **No Change**  **N (%)** | **Consistently No (%)** | **Consistently  Yes (%)** | **Exaggeration**  **(No-Yes)** | **Diminishment**  **(Yes-No)** | **Difference**  **(%)** | **Total** |  |
| **Death of Loved One** | |  |  |  |  |  |  |  |
|  | 148 (55.64%) | 130 (48.87%) | 18 (6.77%) | 65 (24.44%) | 53 (19.92%) | 44.36% | 266 |  |
| **Physical Illness of Loved One** | |  |  |  |  |  |  |  |
|  | 219 (82.33%) | 216 (81.2%) | 3 (1.113%) | 28 (10.53%) | 19 (7.14%) | 17.67% | 266 |  |
| **Personal Physical Illness** | |  |  |  |  |  |  |  |
|  | 245 (92.11%) | 245 (92.11%) | 0 (0%) | 13 (4.89%) | 8 (3.01%) | 7.89% | 266 |  |
| **Major Accident** | |  |  |  |  |  |  |  |
|  | 231 (86.84%) | 230 (86.47%) | 1 (0.38%) | 23 (8.65%) | 12 (4.51%) | 13.16% | 266 |  |
| **War Exposure** |  |  |  |  |  |  |  |  |
|  | 175 (63.64%) | 21 (7.64%) | 154 (56%) | 50 (18.18%) | 50 (18.18%) | 36.36% | 275 |  |
| **Neglect** |  |  |  |  |  |  |  |  |
|  | 190 (77.87%) | 183 (75%) | 7 (2.87%) | 30 (12.3%) | 24 (9.84%) | 22.13% | 244 |  |
| **Hit by Parents** | |  |  |  |  |  |  |  |
|  | 183 (76.57%) | 176 (73.64%) | 7 (2.93%) | 32 (13.39%) | 24 (10.04%) | 23.43% | 239 |  |
| **Sexual Abuse** |  |  |  |  |  |  |  |  |
|  | 231 (92.4%) | 231 (92.4%) | 0 (0%) | 7 (2.8%) | 12 (4.8%) | 7.6% | 250 |  |

Table S4 Simple and Multiple Multinomial Analyses of Each Memory Alteration Patterns: Diminishment and Exaggeration Compared to No Change in Memory

|  | **Diminishment vs No Change in Memory** | | | | | | **Exaggeration vs No Change in Memory** | | | | | |
| --- | --- | --- | --- | --- | --- | --- | --- | --- | --- | --- | --- | --- |
|  | OR | 95% CI | p-value | aOR | 95% CI | p-value | OR | 95% CI | p-value | aOR | 95% CI | p-value |
| **Age** | 1.02 | 0.99-1.04 | 0.154 |  |  |  | 0.99 | 0.97-1.02 | 0.618 |  |  |  |
| **Gender** |  |  |  |  |  |  |  |  |  |  |  |  |
| Female | 1.00 |  |  | 1.00 |  |  | 1.00 |  |  | 1.00 |  |  |
| Male | 0.76 | 0.37-1.56 | 0.451 | 0.63 | 0.26-1.53 | 0.305 | 0.94 | 0.46-1.9 | 0.859 | 0.84 | 0.34-2.04 | 0.699 |
| **Profession** |  |  |  |  |  |  |  |  |  |  |  |  |
| Non-Clinical | 1.00 |  |  |  |  |  |  |  |  |  |  |  |
| Clinical | 1.29 | 0.37-1.56 | 0.443 |  |  |  | 1.00 | 0.37-1.56 | 1 |  |  |  |
| **PTSD (W3)** |  |  |  |  |  |  |  |  |  |  |  |  |
| No | 1.00 |  |  |  |  |  | 1.00 |  |  |  |  |  |
| Yes | 1.34 | 0.7-2.56 | 0.382 |  |  |  | 0.98 | 0.5-1.9 | 0.946 |  |  |  |
| **PTSD (W4)*** |  |  |  |  |  |  |  |  |  |  |  |  |
| No | 1.00 |  |  | 1.00 |  |  | 1.00 |  |  | 1.00 |  |  |
| Yes | 5.80 | 0.7-48.14 | 0.104 | 2.45 | 0.26-23.18 | 0.433 | 18.78 | 2.46-143.33 | 0.005 | 8.04 | 0.98-65.73 | 0.052 |
| **Depression Trend*** |  |  |  |  |  |  |  |  |  |  |  |  |
| Never Depressed | 1.00 |  |  | 1.00 |  |  | 1.00 |  |  | 1.00 |  |  |
| Consistently Depressed | 0.84 | 0.05-13.67 | 0.9 | 0.69 | 0.04-11.71 | 0.797 | 1.87 | 0.16-21.16 | 0.614 | 1.25 | 0.10-16.96 | 0.851 |
| Remitted Depression | 1.25 | 0.42-3.74 | 0.685 | 1.09 | 0.32-3.69 | 0.893 | 1.40 | 0.47-4.19 | 0.574 | 0.95 | 0.27-3.42 | 0.942 |
| Developed Depression | 2.51 | 0.49-12.92 | 0.272 | 2.10 | 0.39-11.21 | 0.385 | 7.93 | 1.75-35.9 | 0.007 | 5.71 | 1.19-27.32 | 0.029 |
| **Psychological Distress Trend*** | | |  |  |  |  |  |  |  |  |  |  |
| Never distressed | 1.00 |  |  | 1.00 |  |  | 1.00 |  |  | 1.00 |  |  |
| Consistently distressed | 4.34 | 0.43-43.86 | 0.213 | 3.51 | 0.33-37.48 | 0.298 | 0.49 | 0.12-1.98 | 0.32 | 0.44 | 0.10-1.96 | 0.28 |
| Remitted distress | 0.60 | 0.03-13.58 | 0.748 | 0.55 | 0.02-12.67 | 0.707 | 0.07 | 0.01-0.82 | 0.035 | 0.08 | 0.01-0.99 | 0.049 |
| Developed distress | 5.08 | 0.5-51.38 | 0.169 | 4.07 | 0.39-42.3 | 0.24 | 0.41 | 0.1-1.67 | 0.214 | 0.31 | 0.07-1.38 | 0.125 |

* Indicates variables that were significant (p-value<0.05) at the simple multinomial level; OR: crude odds ratio of simple multinomial analyses; OR: odds ratio; aOR: adjusted odds ratio of multiple multinomial analyses
